# Genetic study of *Camelina sativa* oilseed crop and selection of a new variety by the bulk method

**DOI:** 10.3389/fpls.2024.1385332

**Published:** 2024-05-28

**Authors:** Martina Ghidoli, Filippo Geuna, Stefano De Benedetti, Sara Frazzini, Michela Landoni, Elena Cassani, Alessio Scarafoni, Luciana Rossi, Salvatore Roberto Pilu

**Affiliations:** ^1^ Department of Agricultural and Environmental Sciences - Production, Landscape and Agroenergy, University of Milan, Milan, Italy; ^2^ Department of Food, Environmental and Nutritional Sciences, University of Milan, Milan, Italy; ^3^ Department of Veterinary Medicine and Animal Sciences, University of Milan, Lodi, Italy; ^4^ Department of Earth and Environmental Sciences, University of Pavia, Pavia, Italy

**Keywords:** camelina, cover crop, oilseed crop, plant breeding, molecular markers

## Abstract

*Camelina sativa*, commonly referred to as camelina or false flax, has emerged as a promising cover crop with the potential to mitigate climate change—a pressing global challenge that demands urgent and sustainable solutions. Belonging to the Brassicaceae family and native to Europe and Central Asia, camelina is an oilseed crop known for its resilience in diverse climates, including arid and semi-arid regions, making it adaptable to various environments. A breeding program started from a study of six winter varieties and five spring varieties of camelina is described: these genetic materials were characterized by SSRs molecular markers and by GBS technique. Molecular data clearly showed all spring varieties were genetically similar and distinguishable from the winter varieties, which, in turn, clustered together. Using molecular data, parental varieties belonging to the two different clusters were selected to generate new genetic variability. The new variety obtained, selected through the bulk method based on three parameters: yield, earliness, and weight of 1000 seeds, has allowed the generation of the new genetic material provisionally named C1244. Chemical characterization was performed (bromatological and glucosinolates analysis) to better describe C1244 in comparison with benchmark varieties. The new variety exhibited early maturity, similar to spring varieties, making this genetic material promising for use in intercropping systems, a high weight of 1000 seeds (1.46 g) which improves and facilitates seeding/harvesting operations and a high oil content (33.62%) akin to winter varieties making it valuable for human and animal food purposes.

## 1 Introduction

Camelina, scientifically known as *Camelina sativa* (L.) Crantz, is an ancient oilseed crop belonging to the Brassicaceae family, specifically to the tribe Camelineae. Also known as false flax or gold of pleasure, this crop has historical roots dating back to the Iron Age and was extensively cultivated in European countries and Russia, its center of origin. However, its cultivation declined after the Second World War due to the emergence of more profitable crops (Ghidoli et al., 2023a).

Characterized by average height ranging from 0.3 to 0.9 m, camelina plants feature branched stems and alternate lanceolate leaves. Its inflorescences form racemes comprising small yellow flowers with four petals, and its smooth, leathery siliques typically contain 5–15 golden/brown seeds, each measuring 2 to 3 mm in length. The weight of 1000 seeds ranges from 0.8 to 2.0 g (Government of Canada, 2012). Camelina stands out as a resilient plant with a rapid growth cycle of approximately 85–100 days for the spring varieties and 190–210 days for winter varieties, making it suitable for various low-input agronomic systems, especially in the context of climate change (Ahmad et al., 2022).

Camelina proves beneficial in intercropping and rotation systems, particularly in arid regions (Berti et al., 2016; Neupane et al., 2022). Winter genotypes stand out as optimal choices for winter cultivation, providing soil protection. Incorporating winter camelina as a cover crop serves to prevent erosion, fosters carbon sequestration in the soil, and additionally functions as a weed control measure by inhibiting their growth (Zubr, 2003; Sydor et al., 2022; Ghidoli et al., 2023b).

Regarding this, in recent years, great attention has been paid to brassicaceous species, including camelina, as cover crops with allelopathic activity (Ghidoli et al., 2023b; Pascual et al., 2024). Some studies report field experiments to study the effects of camelina used as a cover crop in rotation with maize, highlighting a reduction in root disease and greater growth and yields compared to maize crops which followed other cover crops such as rye (Acharya et al., 2020).These studies suggest potential benefits for using camelina as a cover crop before growing corn. The positive effects of cover crops on soil quality, especially in low-carbon or degraded soils and soil structure, prevention of erosion and mitigation of crop diseases nowadays are being widely studied (Acharya et al., 2020). Furthermore, there has been a surge of interest in this crop due to its versatile applications, serving as a new source of polyunsaturated fatty acids and proteins for feed, food, and bio-based products (Hajiazizi et al., 2024). Camelina oil is particularly attractive for its high oil content in seeds (up to 40%) and its substantial proportion of unsaturated fatty acids, including 30–40% alpha-linolenic acid, 15–25% linoleic acid, 15% oleic acid, and about 15% eicosenoic acid (Rodríguez-Rodríguez et al., 2021).

Despite its nutritional benefits, camelina oil and the protein cake derived from seed pressing contain antinutritional compounds such as glucosinolates, synapin, phytic acid, condensed tannins, and erucic acid (Russo and Reggiani, 2012). Among these, glucosinolates (GSL) pose a significant limitation, especially in feed applications (Murphy, 2016).

In this scenario, when selecting new varieties of camelina it is necessary to consider the main agronomic characteristics, including yield (expressed in seed size and competitive ability), earliness, and the reduction of antinutritional compounds such as glucosinolates. Breeding programs face challenges due to camelina’s allohexaploid nature (chromosome number 2n = 40, genome size 750 Mbp) and limited genetic variability (Ghidoli et al., 2023a; Blume et al., 2023). However, some goals were achieved, in fact Lolli and colleagues, through classical breeding using the pedigree method obtained an improved camelina line with lower glucosinolates content which allowed the incorporation of up to 20% camelina cake in the diet of laying hens without any adverse effects on animal welfare and health, eggshell quality, or production performance (Lolli et al., 2020). Recent findings in genomics studies are promising a great contribution to improve camelina by the use in the selection scheme of Marker-Assisted Breeding (Li et al., 2021).

In this work we present data on a breeding program involving crossing between two varieties selected on the basis of molecular data carried out from a characterization performed with molecular markers (SSRs) and subsequently by GBS (Genotyping by Sequencing) technique that helped us to choose the parental lines. The resulting new variety, named C1244, shows interesting traits coming from the two different genetic pools associated with spring and winter varieties used in this work.

## 2 Materials and methods

### 2.1 Plant materials

Six winter lines, five spring lines and a new variety of camelina generated by crossing a spring and a winter variety (**Table 1**) were tested in an experimental field of the University of Milan, in Landriano (PV) (45°19′N 9°16′E), 88 m a.s.l. (North Italy) by low input farming.

**Table 1:** *Camelina sativa* genotypes under study.

ID CODE	Name varieties	Genetic constitution
C1232	Experimental material	Winter pure line
C1233	Experimental material	Winter pure line
C1234	Experimental material	Winter pure line
C1235	Experimental material	Winter pure line
C1236	Experimental material	Winter pure line
C1237	Experimental material	Winter pure line
C1238	Experimental material	Spring pure line
C1239	Experimental material	Spring pure line
C1240	Calena	Spring pure line
C1241	Madalina	Spring pure line
C1243	Omich	Spring pure line
C1244	New variety (C1243 x C1233)	Spring inbred line

The study aimed to carry out an agronomic comparison of the types of varieties (spring and winter) of camelina and an evaluation of the new variety developed, deriving from the crossing of the two types of lines, during winter cultivation in North Italy. The varieties Omich and Madalina are registered in the Community Plant Variety Office (CPVO) and the variety Calena was kindly provided by Dr. Galasso Incoronata, IBBA-CNR of Milan (Italy). The other experimental lines are provided by the germplasm bank at the Department of Agricultural and Environmental Sciences – Production, Landscape and Agroenergy (DISAA), University of Milan, Italy.

### 2.2 Molecular analyses

#### 2.2.1 Microsatellite analysis

The molecular analysis was performed on the DNA extracted from the leaves of the genetic materials (**Table 1**) using the molecular markers SSRs (**Supplementary Table 1**).

Genomic DNA was extracted from 1g of plant tissue, following the Steve1 method modified. The plant material was homogenized in a mortar with liquid nitrogen and transferred to centrifuge tubes, into which 3mL of extraction buffer (EB1) was added. After shaking the tubes, 3mL of Phenol: Chloroform 1:1 was added and centrifuged for 20 minutes at 12000 rpm at 4°C. The sample was separated into two phases, the supernatant was recovered, and isopropanol was added and centrifuged for 30 minutes at 10000 rpm at 4°C, the pellet was recovered, allowed to air dry, and then 500μL of distilled water was added. After resuspending the pellet, the solution was transferred to an Eppendorf tube. A second precipitation was performed by adding 200μL of 3M CH3COONa and 700μL of isopropanol; the solution was shaken and kept on ice for 15 minutes, after which it was centrifuged for 30 minutes at 13000 rpm. Afterwards, the supernatant was removed, and the pellet was resuspended in 50μL of distilled water with 3μL of Rnase (10μg/mL). The extracted DNA was diluted 5 times, and 1µL was used for Polymerase Chain Reactions (PCR). PCR were carried out as described in Manca et al. (2013) by using labeled primers with 6-FAM. Sizing was performed in outsourcing by BMR genomics (Padova, Italy). PCR reactions were performed in a thermocycler, where the amplification conditions consisted of an initial denaturation cycle at 94°C for 5’ followed by 35 denaturation cycles for 30’’ at 94°C, primer annealing at the reported annealing temperature in **Supplementary Table 1** (Ta) for 30’’ and extension for 30’’ at 72°C, with a final extension cycle for 30’ at 72°C. TD1 and TD2 are two slightly different touchdown PCR protocols. TD1 consisted in an initial 5’ denaturation phase at 94°C, followed by 10 cycles at 94°C of 30’’, 30’’ annealing phase at 65°C reduced by 1°C each cycle and 30’’ at 72°C. This first series of cycles was followed by 30 cycles at 94°C for 30’’, at 55°C for 30’’, at 72°C for 30’’ and a final extension phase at 72°C for 30’. TD2 instead differed from TD1 in the annealing temperature which started from 54°C and was reduced by 0.5°C for 8 cycles which were followed by 32 cycles at 50°C (Manca et al., 2012). By electrophoresis, the amplified fragments were fractionated using 4% (w/v) agarose gel and stained with ethidium bromide. The amplifiers were outsourced to BMR Genomics for sizing. The results obtained were used to compile the matrix used to generate the dendrogram obtained with the UPGMA (Unweighted Pair Group Method Using Arithmetic Averages) algorithm and the PCA (Principal Coordinate Analysis) analysis using the GenAlEx 6.5 program.

#### 2.2.2 Genotyping by sequencing analysis

Library preparation and sequencing (Paired-End mode, Illumina platform) were done by the Biodiversa (Rovereto, Italy) company. Upon receipt of FASTQ data files, a preliminary quality control was done using the NGSEP (v.4.01) software (Tello et al., 2019).

The camelina reference genome used for the downstream analyses was retrieved at https://plants.ensembl.org/Camelina_sativa/Info/Index. Prior to mapping, an index of the reference genome was obtained through the Bowtie v.2.4.3 software (Langmead and Salzberg, 2012). Reads were finally mapped against the reference using the ‘ReadsAligner’ module of the NGSEP software. Mapped reads were sorted by position in the reference genome using the Picard v.2.26.3 software (Picard, 2021). Single nucleotide polymorphism (SNP and InDel) variants were detected using the NGSEP module ‘SingleSampleVariantsDetector’ with the flag ‘-maxAlnsPerStartPos = 100’ (suggested for GBS or RAD analyses) and the VCF annotate module. The obtained variant calling files (VCFs) were employed for the creation of the distance matrix between samples and the construction of the dendrogram using the NGSEP modules ‘VCF_distance_matrix’ and ‘neighbor_joining’, respectively. The output of the cluster analysis was a file in the Newick format (https://phylipweb.github.io/phylip/newicktree.html). For all the analyses the high-performance computing (HPC) cluster at the University of Milano (http://www.indaco.unimi.it) (INDACO HPC cluster, 2024) was employed running under a CentOS 7 operating system with a minimum of 16 cores and 64 GB RAM for the most demanding runs of index building and read mapping.

### 2.3 Breeding program

The new variety C1244 was developed starting with a cross performed in 2018, between C1243 Omich CPVO variety (spring-type) and C1233, an experimental material of the University of Milan (winter-type). The breeding program used the bulk method of selection, in which following the initial 10 crossings (about 100 F1 plants), it obtained about 10,000 plants in the F2 generation. Subsequently, in each next generation (for 5 propagation cycles) about 15,000 seeds were collected and propagated. Generational advances were conducted through propagation cycles both in greenhouses and in the field (2 to 3 generations per year). About 50 plants were selected from the synthetic population in F6, evaluated by progeny-row and the inbred line deemed best was selected.

### 2.4 Field trials

The experimentation was conducted for two years in the experimental field of the University of Milan in Landriano (PV). Agronomic comparisons were carried out by evaluating the commercial winter-type and the spring-type pure lines of camelina in two different agronomic seasons, 2021/22 and 2022/23. In the first year, the camelina sowing was conducted on 14 October 2021 and the harvest on 31 May 2022, while, in the second year, the sowing was carried out on 27 October 2022 and the harvest on 16 June 2023. The two field studies were conducted in low-input cultivation without irrigation throughout the field. Neither agrochemicals nor herbicides were used to defend the crop and no fertilization was carried out as the soil appears to be rich in organic substance as reported in the analyses in Landoni et al. (2020). **Supplementary Table 2** presents the average temperature (°C) and rainfall (mm) data for the relevant months in the two experimental fields. For both years, the experimentation was laid out in randomized blocks in which each accession (12 total materials) was cultivated in three plots each, measuring approximately 6 m^2^ (1.2 m x 5 m), for a total of 216 m^2^. Seeding was carried out on tilled soil using an Earthway 1001B manual seeder, with 1002–5 seeding disc, in rows spaced 0.2 m, at a seed density of 8 kg/ha.

Several observations were carried out during the crop cycle. The days from sowing to flowering were recorded in each plot, when 50% of the plants had at least one open flower. Once the plants matured, all the plots were harvested manually by cutting the plants at ground level. The harvested plants were stored in a dry room to complete the drying process, and at the end, the seeds were collected by manually opening the siliques and by sifting.

### 2.5 Seed quality analysis

The analysis was performed by aggregating 100 g of seeds per plot for each variety. Subsequently, a 5 g sample was selected from the pooled seeds for bromatological analysis. Camelina seeds underwent an assessment in triplicate for essential nutrients, including dry matter (DM), ashes (A), oil (O), crude proteins (CP), and crude fiber (CF), following the procedures outlined in the AOAC (Association of Official Agricultural Chemists) guidelines of 2019. The determination of DM involved desiccating seeds in a forced air oven at 65°C for 24 hours (AOAC method 930.15). Ashes were acquired by incinerating samples in a muffle furnace at 550°C (AOAC method 942.05). O was ascertained using ether extraction in a Soxtec system (SER 148 Series Solvent Extractor, Velp Scientifica Srl, Usmate, Italy; AOAC method 2003.05). CPs were calculated through the Kjeldahl method (AOAC method 2001.11). For CF, the determination was carried out using the filter bag method (AOCS method Ba 6a-05) (AOCS, 2009).

### 2.6 Glucosinolates analysis

To perform glucosinolates (GLS) quantification, seeds were ground in liquid Nitrogen with mortar and pestle. To avoid endogenous myrosinase activity all samples were continuously kept frozen and stored at -80°C until analysis. To extract GLS, milled seeds were suspended in 5 ml of 80% Methanol (Sigma-Aldrich) 1:25 ratio and incubated for 30 minutes at 70°C. Extract was centrifuged at 9,000 rpm for 20 minutes and 4 ml of the resulting supernatant was transferred. The solvent was removed under vacuum with an Eppendorf concentrator plus (Eppendorf). To perform GLS quantification the obtained material was resuspended in 1 ml of 50 mM Citrate Buffer pH 6. Enzymatic reaction was performed on 200 µl of centrifuged supernatant to which was added 0.3 U of Thioglucosidase from *Sinapis alba* (white mustard) seed (Sigma-Aldrich) in a final volume of 500 µl at 25°C for 20 min. Glucose released by Thioglucosidase from Glucosinolate was measured with Enzytec™ Generic D-Glucose/D-Fructose/Sucrose kit (R-biopharm) following manufacturer’s instructions. Free glucose quantification was performed for each sample without the addition of Thioglucosidase as negative control; the obtained value was subtracted from the relative glucose quantification. Each experiment was performed in duplicate.

### 2.7 Informatic tools

The program used for data collection was Microsoft Excel^®^ and the one to perform the statistical analysis was PAST (Paleontological Statistics, version 4.12). Results are shown as standard deviation of least squares means, and statistically significant differences are accounted for p ≤ 0.05. The GenAlEx 6.5 program was used for the analysis of molecular data.

## 3 Results

### 3.1 Genetic characterization using SSR molecular markers and GBS analysis

With the aim of developing a new variety of camelina, different genetic materials (**Table 1**) were collected and cultivated in the experimental field of the University of Milan. The six winter-types and the five spring-types varieties were studied using 16 molecular markers (SSR) (**Supplementary Table 1**) (Manca et al., 2012).

**Figure 1:**
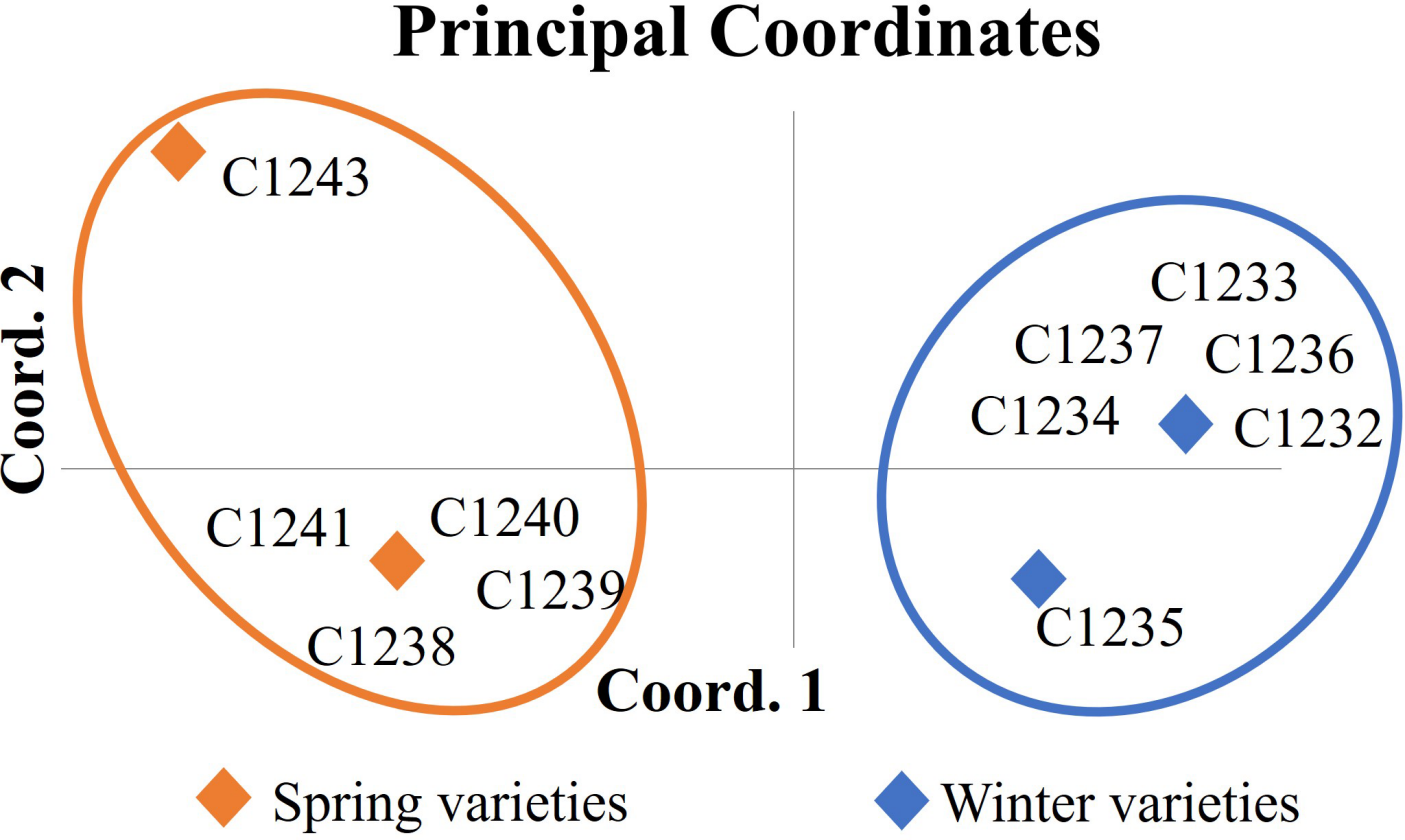
Principal coordinate analysis (PCA) of *Camelina sativa* varieties based on 16 molecular markers. In orange cluster spring varieties (C1238, C1239, C1240, C1241 and C1243) and in blue cluster winter varieties (C1232, C1233, C1234, C1235, C1236 and C1237).

The genetic matrix obtained after amplicon sizing was elaborated by the GenAlEx program with the aim to assess genetic differences among varieties collected. The dendrogram obtained by UPGMA (Unweighted Pair Group Method Using Arithmetic Averages) method showed the same result found with the PCA analysis (Principal Coordinate Analysis) (**Figure 1**): two different clusters with the two germplasm-types, the spring varieties (C1238, C1239, C1240, C1241 and C1243) and the winter ones (C1232, C1233, C1234, C1235, C1236 and C1237).

**Figure 2:**
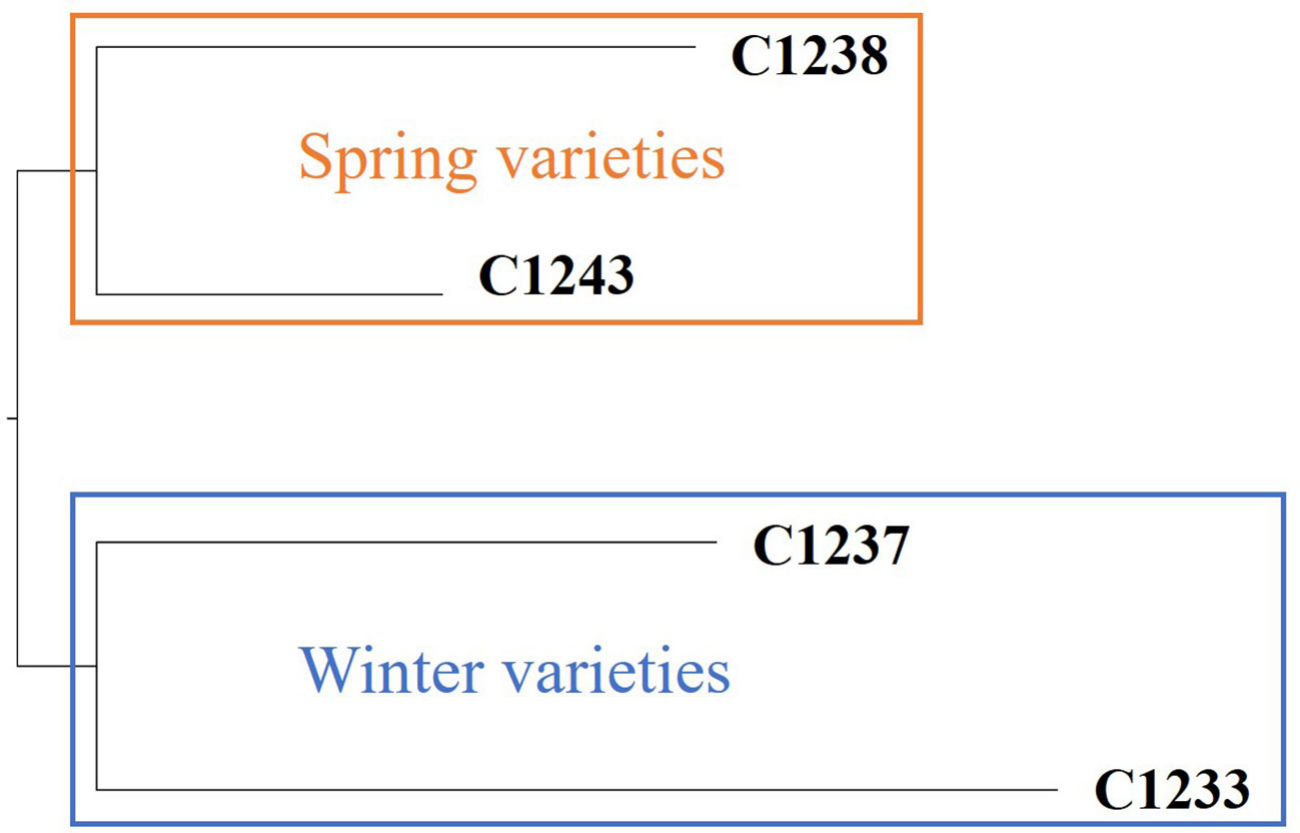
Dendrogram resulting by GBS analysis. In orange cluster spring varieties and in blue cluster winter varieties.

To validate the outcomes achieved through SSRs, a more comprehensive genetic analysis was conducted using the Genotyping by Sequencing technique. Based on a total of 78,863 variants detected among four samples, corresponding to two spring varieties and two winter varieties, it is possible to confirm the genetic distinctiveness between the two camelina seasonal behaviors. In particular, the highest relative distance (0.231) is observed between spring variety C1238 and winter variety C1233 (**Figure 2**).

### 3.2 Parental selection and breeding program

**Figure 3:**
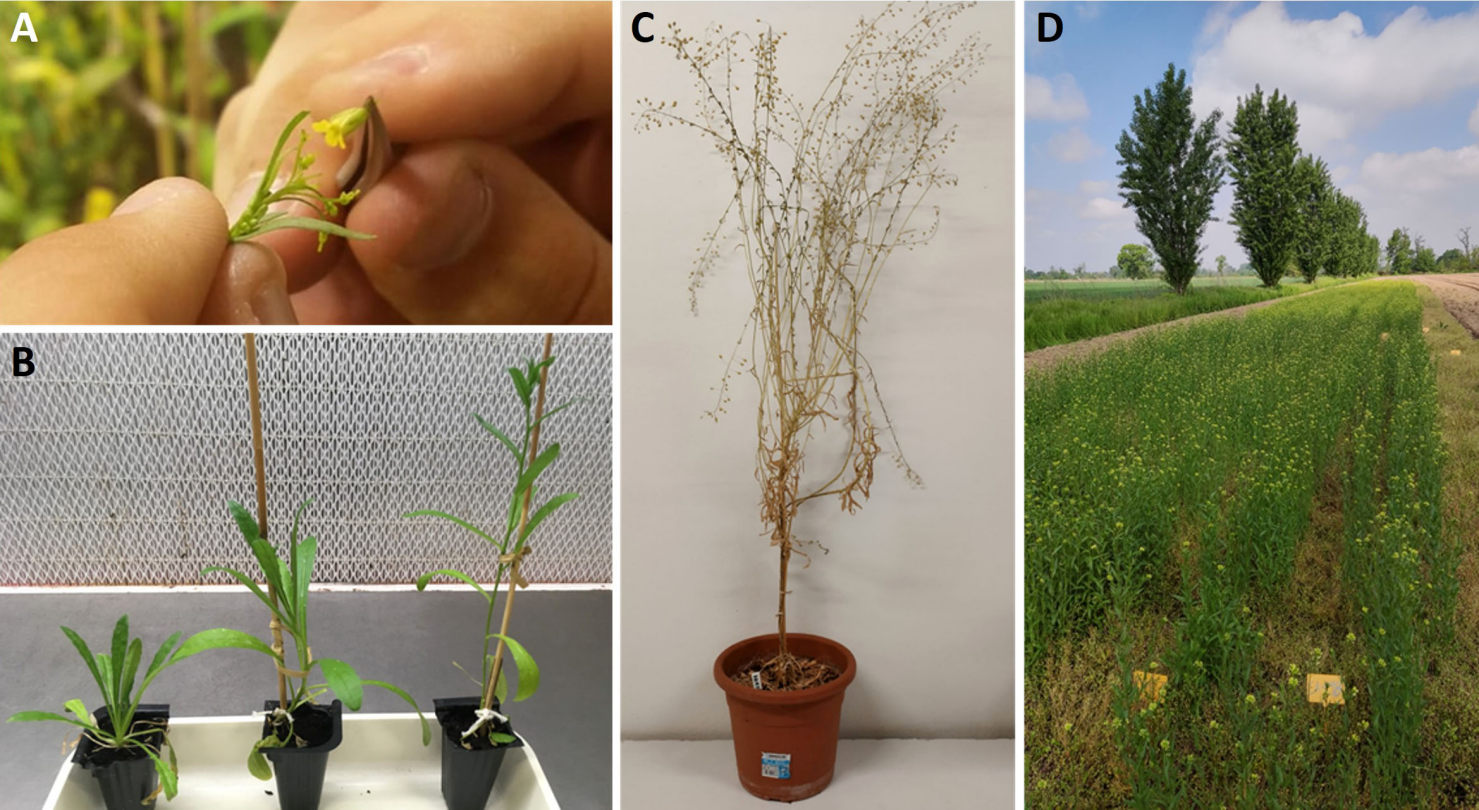
**(A)** *Camelina sativa* crossing. **(B)** On the left, camelina winter variety; on the right, camelina spring variety; in the middle, F1 obtained. **(C)** F1 plant at maturity. **(D)** Camelina experimental field.

The selection of the parental lines was carried out by evaluating molecular data reported in the previous section (3.1 Genetic characterization using SSR molecular markers and GBS analysis), and also considering the agronomic traits: yield, earliness and weight of 1000 seeds (data not shown). Merging agronomic and molecular data, it was therefore decided to cross the spring variety C1243 (commercial variety Omich) and the winter experimental variety C1233 of the University of Milan (**Figure 3**). The new variety C1244 (spring-type variety) was developed by a breeding program employing the bulk selection method as reported in the Materials and Methods. In the F6 generation, the selected plants were evaluated on the base of the traits yield, earliness and weight of 1000 seeds and the best-performing inbred line (C1244) was multiplied with the aim to perform agronomic comparisons.

### 3.3 Agronomic comparison

The new variety developed, C1244 was compared to the benchmarks concerning agronomic traits and propagated in the open field for two consecutive years. The experimental setup utilized randomized blocks, with each genetic line cultivated as detailed in the Materials and Methods section. Both experimental fields were sown in October and harvested at the end of May. Yield (Y), number of plants per m^2^ (NP) and weight of 1000 seeds (W1000) were the agronomic characteristics assessed for each genetic material.

The two-way ANOVA performed on the data collected for the Y and the W1000 data showed statistically significant differences considering two years of trials for the factors variety and year. For what concern interaction between variety and year, we observed statistically significant differences only for Y. Regarding NP trait, in the analysis the variety factor is not statistically significant, while year and interaction factors were (**Table 2**).

**Table 2:** Two-way ANOVA regarding the traits: yield (Y), number of plants per m^2^ (NP) and weight of 1000 seeds (W1000) collected on all the varieties (ID CODE) in the two years trials.

Traits		Sum of sqrs	df	Mean square	*F*	*p* (same)
Y	**ID CODE:**	1,1375E07	11	1.03409E06	7,531	2,532E-07
	**YEAR:**	1,09828E07	1	1,09828E07	79,98	8,598E-12
	**Interaction:**	3,25948E06	11	296317	2,158	0,0334
	**Within:**	6,59128E06	48	137318		
	**Total:**	3,22085E07	71			
NP	**ID CODE:**	23357,3	11	2123.39	1,467	0,1753
	**YEAR:**	515620	1	515620	356,3	7,489E-24
	**Interaction:**	82718	11	7519,82	5,196	2,536E-05
	**Within:**	69464,7	48	1447,18		
	**Total:**	691160	71			
W1000	**ID CODE:**	0,6163	11	0,0560273	13,21	4,474E-11
	**YEAR:**	0,0882	1	0,0882	20,79	3,542E-05
	**Interaction:**	0,0535	11	0,00486364	1,147	0,3483
	**Within:**	0,2036	48	0,00424167		
	**Total:**	0,9616	71			

Tukey’s test post-hoc analysis is showed in **Supplementary Tables 3, 4** and **5**.

**Figure 4:**
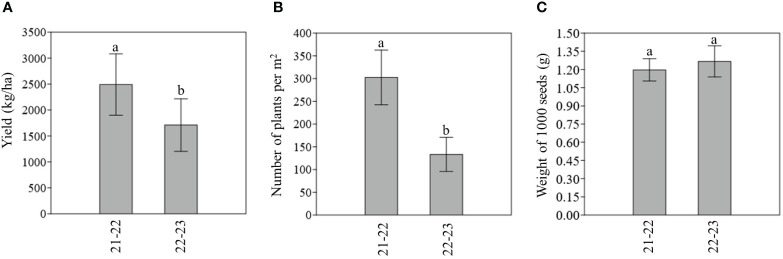
**(A)** Average estimated yield (kg/ha) per year. **(B)** Average number of plants per m^2^ per year. **(C)** Average weight of 1000 seed (g) per year. For each parameter measured, different letters indicate statistically significant differences (Tukey’s test, p < 0.05). Bar represents the standard deviation.

Considering the two years of trials separately, as shown in **Figure 4**, we can observe that on average camelina Y and NP were statistically higher in the first year (2021–2022) (**Figures 4A, B**). No statistically significant difference were observed for W1000 (**Figure 4C**).

As reported in **Table 3**, concerning Y, in the season 2021–2022 the highest value was recorded by the winter parental line C1233 (3392.5 ± 382), while the lowest was obtained by the spring parental line C1243 (1670.2 ± 132). The new variety C1244 recorded a yield of 2160.3 kg/ha. In the second year, the most productive variety was always C1233, with a yield of 2605 kg/ha, the least productive was C1232 (900.3 ± 140), while C1244 recorded the second-highest yield (2173.3 ± 335) (**Table 3**).

**Table 3:** Agronomic characterization of *Camelina sativa* benchmark cvs (C1232, C1233, C1234, C1235, C1236, C1237, C1238 C1239, C1240, C1241 and C1243) and the new variety (C1244) in two years of winter cultivation.

ID CODE	Y (kg/ha)		NP/m^2^		W1000 (g)	
	2021–2022	2022–2023	2021–2022	2022–2023	2021–2022	2022–2023
**C1232**	1975.2 ± 140 ^bc^	900.3 ± 140 ^b^	288.3 ± 66.6 ^ab^	133.3 ± 17.6 ^ab^	1.12 ± 0.05 ^cd^	1.12 ± 0.01 ^cd^
**C1233**	3392.5 ± 382 ^a^	2605 ± 585 ^a^	184.3 ± 23.1 ^b^	201.7 ± 33.3 ^a^	1.09 ± 0.04 ^d^	1.16 ± 0.04 ^cd^
**C1234**	2949.3 ± 920 ^ab^	1908.3 ± 393 ^a^	296.7 ± 35.5 ^ab^	158.3 ± 58 ^ab^	1.14 ± 0.09 ^bd^	1.10 ± 0.13 ^d^
**C1235**	2784.3 ± 353 ^ac^	1375 ± 51 ^b^	262.5 ± 12.5 ^ab^	140 ± 32.8 ^ab^	1.12 ± 0.07 ^bd^	1.22 ± 0.05 ^bd^
**C1236**	2700.8 ± 492 ^ac^	1605 ± 400 ^b^	355 ± 15.2 ^a^	163.3 ± 42.5 ^ab^	1.13 ± 0.07 ^bd^	1.20 ± 0.07 ^bd^
**C1237**	2558.1 ± 562 ^ac^	2010 ± 180 ^a^	285.2 ± 10.1 ^ab^	130 ± 18 ^ab^	1.21 ± 0.03 ^bd^	1.22 ± 0.03 ^bd^
**C1238**	2401.8 ± 365 ^ac^	1888.3 ± 621 ^a^	320 ± 77.6 ^a^	93.3 ± 17.6 ^b^	1.24 ± 0.08 ^ad^	1.33 ± 0.04 ^abc^
**C1239**	1921.8 ± 212 ^bc^	1557.5 ± 42.5 ^b^	328.3 ± 35.1 ^a^	113.3 ± 32.1 ^b^	1.28 ± 0.03 ^ab^	1.39 ± 0.06 ^ab^
**C1240**	2933.5 ± 610 ^ac^	1641.7 ± 255 ^b^	321.7 ± 79.4 ^a^	117.5 ± 7.6 ^ab^	1.23 ± 0.03 ^ad^	1.29 ± 0.11 ^ad^
**C1241**	2448.5 ± 40 ^ab^	1438.3 ± 73 ^b^	377.5 ± 37.6 ^a^	115 ± 5.2 ^b^	1.25 ± 0.03 ^abc^	1.41 ± 0.13 ^ab^
**C1243**	1670.2 ± 132 ^c^	1420 ± 35 ^b^	298.3 ± 46.5 ^ab^	120 ± 31.2 ^ab^	1.16 ± 0.05 ^bd^	1.30 ± 0.07 ^ad^
**C1244**	2160.3 ± 373 ^bc^	2173.3 ± 335 ^a^	312.5 ± 22.7 ^ab^	113.3 ± 2.9 ^b^	1.37 ± 0.02 ^a^	1.46 ± 0.05 ^a^
**Total**	**2491.3 ± 591**	**1710.2 ± 507**	**302.5 ± 60.1**	**113.3 ± 37.4**	**1.20 ± 0.09**	**1.27 ± 0.13**

Parameters include Yield (Y), number of plants per m^2^ (NP/m^2^) and weight of 1000 seeds (W1000). For each parameter measured, the statistic is presented in columns, different letters indicate statistically significant differences (Tukey’s test, p < 0.05).

Regarding NP, the variety with the highest number of plants in the first year was C1241 (377.5 ± 37.6), while in the second year it was C1233 (201.7 ± 33.3) which, however, in the first year was the variety that had the lowest NP (184.3 ± 23.1). The new variety C1244 recorded an NP almost three times higher in the first year than in the second year (312.5 ± 22.7 and 113.3 ± 2.9) (**Table 3**).

**Figure 5:**
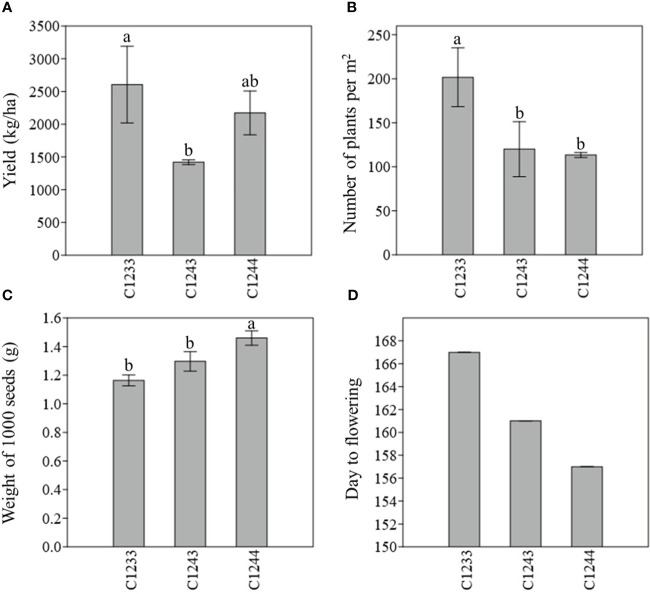
**(A)** Estimated yield (kg/ha) **(B)** Number of plants per m^2^ **(C)** Weight of 1000 seeds (g) **(D)** Earliness (day to flowering); field 2022–2023; C1233 winter parental line; C1243 spring parental line; C1244 new variety. For the first three parameter measured, different letters indicate statistically significant differences (Tukey’s test, p < 0.05). Bar represents the standard deviation.

With regard to W1000, the variety that recorded the highest weight in both years was the new variety C1244 (1.37 ± 0.02 and 1.46 ± 0.05) which was found to be statistically different (p < 0.05) particularly from all the winter varieties. Meanwhile, the one with the lowest W1000 was in the first year (2021–2022) the parental line C1233 (1.09 ± 0.04), and in the second year (2022–2023), the variety C1234 (1.10 ± 0.13) (**Table 3**). As described before, the new variety was obtained by selecting for Y, NP, W1000 and earliness, the data collected for these traits are summarized in **Figure 5** for the two parental lines C1233 and C1243 and for the new variety C1244.

**Figure 6:**
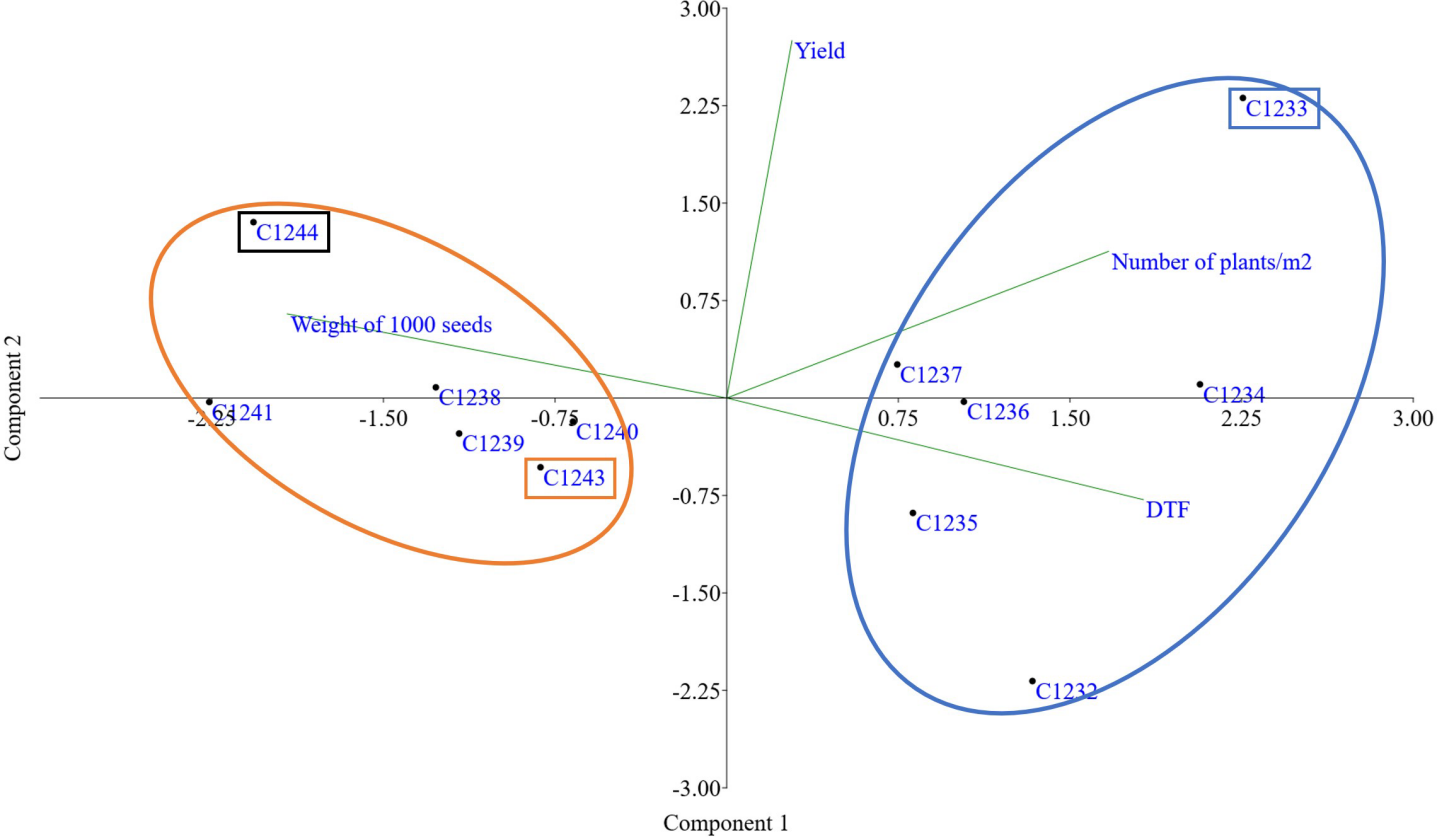
PCA obtained using yield, number of plants, weight of 1000 seeds (g) and earliness (Day to flowering). In orange cluster spring varieties and in blue cluster winter varieties. In orange rectangle spring parental line (C1243), in blue winter parental line (C1233) and in black new variety (C1244).

Furthermore, a PCA multivariate analysis was conducted on all the varieties under study, considering the four agronomic parameters by imposing k means equal to 2 the two clusters defined by the molecular analysis were found: the cluster composed of winter varieties C1232, C1233, C1234, C1235, C1236 and C1237 and the cluster with the spring varieties C1238, C1239, C1240, C1241, C1243 and C1244 (**Figure 6**).

### 3.4 Analysis of nutritional composition and glucosinolates levels

**Figure 7:**
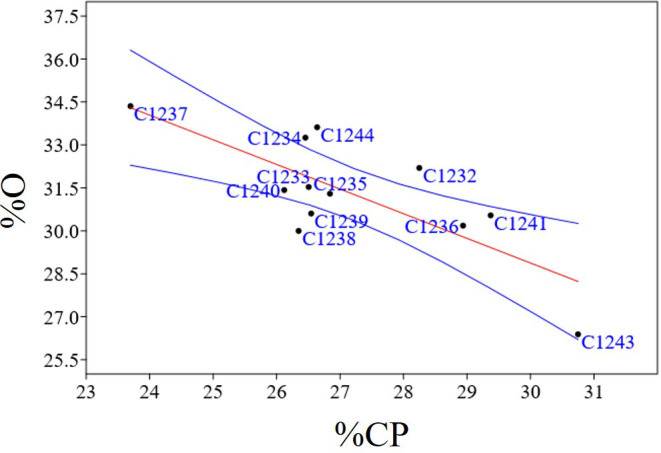
Regression plots of statistically significant negatives correlation between %O and %CP (r = - 0.76; p = 0.0038); In blue confidence band for the regression, in red regression line.

**Figure 8:**
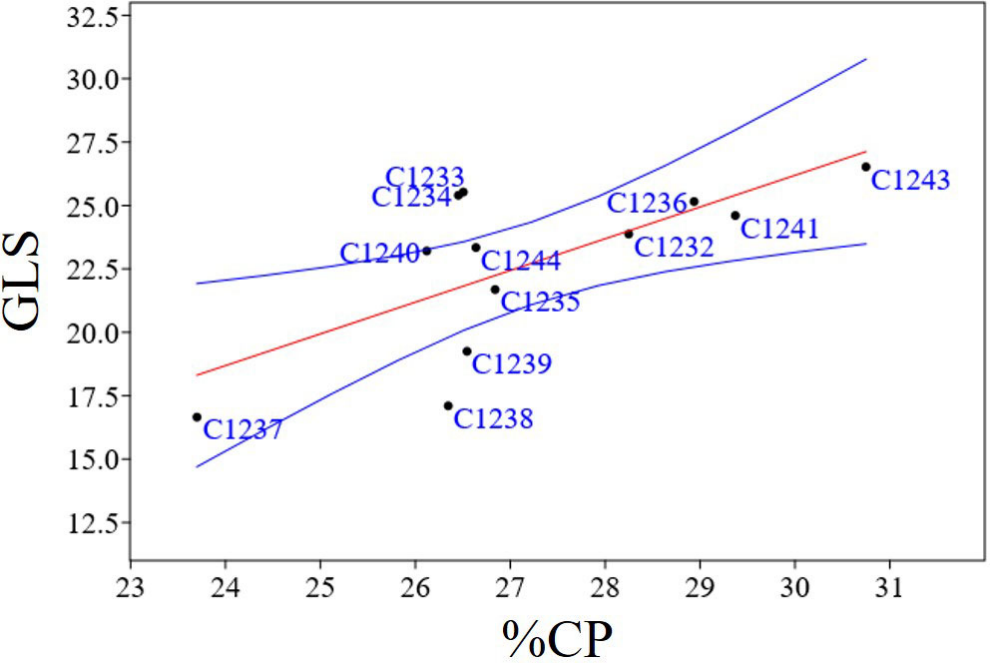
Regression plots of statistically significant positive correlation between GLS and %CP (r = 0.69; p = 0.013); In blue confidence band for the regression, in red regression line.

**Figure 9:**
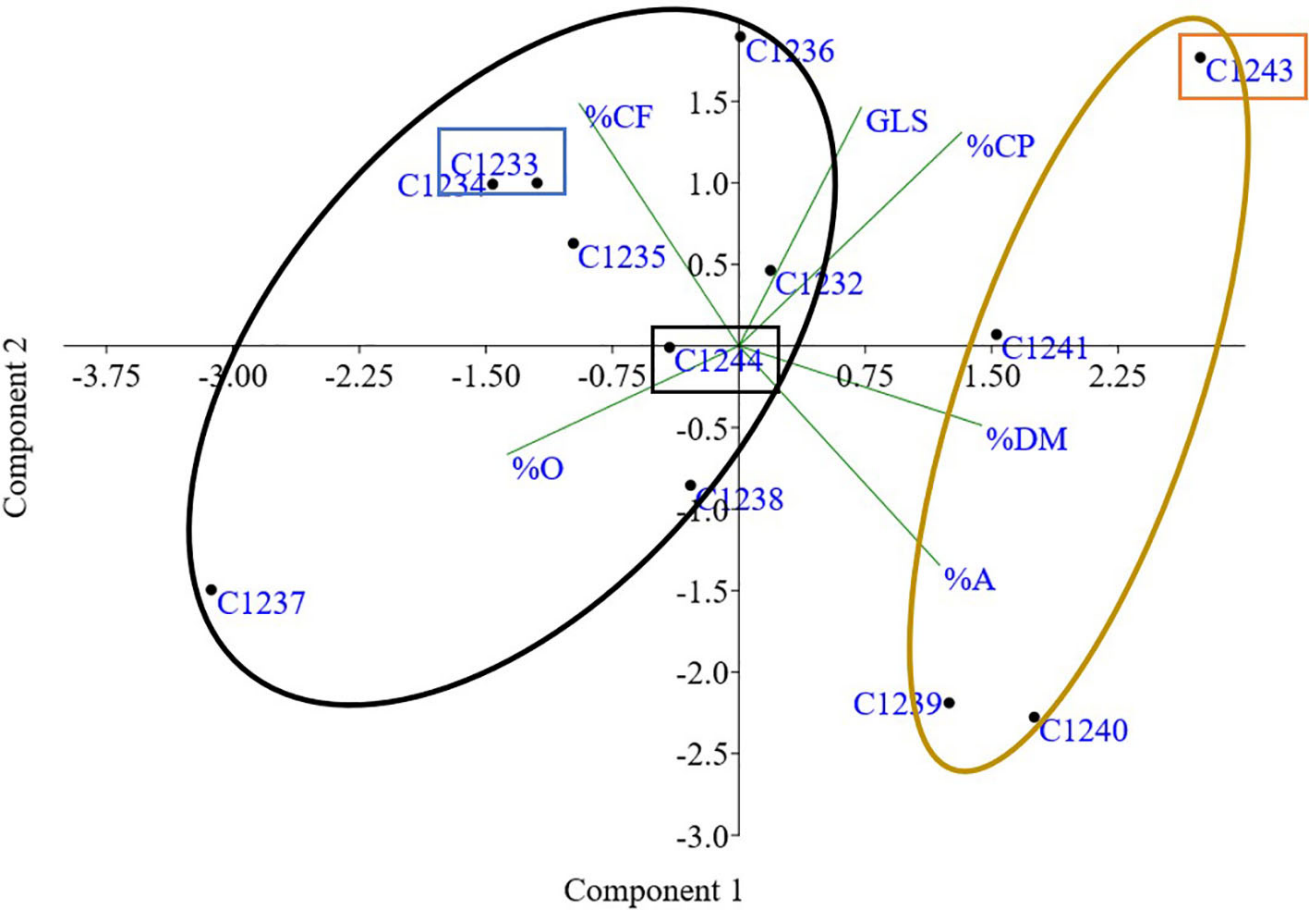
PCA obtained using bromatological traits and glucosinolates content. Two clusters obtained by imposing k means = 2; In orange rectangle spring parental line (C1243), in blue winter parental line (C1233) and in black new variety (C1244). Dry matter (%DM), ash (%A), crude fiber (%CF), protein (%CP), oil (%O) and glucosinolates (GLS).

To better characterize the new variety obtained, bromatological analysis on dry matter (%DM), ash (%A), crude fiber (%CF), protein (%CP) and oil content (%O) and the glucosinolates (GLS) quantification were performed on the benchmark cultivars and on the C1244 variety in the season 2022–2023 (**Table 4**). As reported in **Table 4**, for the %DM, no statistically significant differences were found among all the varieties studied (seed residual moisture at harvest was around 5% for all the varieties), while for all the other traits there were differences. Regarding in particular %O and %CP, the variety C1237 recorded the highest %O (34.35 ± 1.37), and the lowest %CP (23.70 ± 1.20). Meanwhile, the lowest %O was recorded by the spring parental line C1243 (26.39 ± 0.78) that registered also the highest %CP (30.75 ± 1.26). The new variety C1244 recorded the second highest %O (33.62 ± 0.20) and the content of %CP was 26.64 ± 1.70 (**Table 4**). A strong negative correlation was found between %O and %CP (r = - 0.76; p = 0.0038) as reported in **Figure 7**. Furthermore, in general for these two main bromatological parameters %CP and %O, the average %CP in winter varieties stood at 26.78% and in spring varieties it was 27.83%. As for the %O average, winter varieties exhibited 32.14%, while spring varieties showed 29.79%. Concerning GLS, the average content in winter varieties was 23.05%, in spring varieties it was 22.14%, and for the new variety (C1244), it was 23.34%. Moreover, the C1237 winter variety stands out for having the lowest amount (16.7 ± 1), whereas the C1243 spring parental variety exhibits the highest content (26.5 ± 0.5) (**Table 4**). A positive correlation was reported in **Figure 8** between GLS and %CP (r = 0.69; p = 0.013). A multivariate analysis was carried out on the parameters of **Table 4** and by imposing k means equal to 2 the two clusters found were: the first with all the winter varieties (C1232, C1233, C1234, C1235, C1236, C1237) and two spring varieties (C1238 and C1244) and the second with the spring varieties C1239, C1240, C1241 and C1243 (**Figure 9**).

**Table 4:** Compositional assessments conducted on *Camelina sativa* seeds, with nutrient composition presented on a dry matter (DM) basis.

ID CODE	%DM	%CF	%A	%CP	%O	GLS (mmol/kg)
**C1232**	95.40 ± 0.54 ^a^	23.07 ± 1.26 ^abd^	4.09 ± 0.03 ^de^	28.25 ± 1.04 ^ab^	32.19 ± 1.25 ^ab^	23.9 ± 0.7 ^b^
**C1233**	94.56 ± 0.91 ^a^	24.28 ± 0.71 ^a^	4.11 ± 0.05 ^de^	26.51 ± 1.55 ^bc^	31.53 ± 1.68 ^ab^	25.5 ± 3.3 ^ab^
**C1234**	94.86 ± 0.74 ^a^	25.39 ± 0.89 ^a^	4.09 ± 0.02 ^de^	26.45 ± 1.34 ^bc^	33.26 ± 0.21 ^ab^	25.4 ± 1 ^ab^
**C1235**	95.07 ± 0.65 ^a^	24.34 ± 1.32 ^ab^	4.00 ± 0.05 ^e^	26.84 ± 0.57 ^bc^	31.29 ± 0.44 ^ab^	21.7 ± 0.1 ^bc^
**C1236**	95.06 ± 0.43 ^a^	25.56 ± 1.81 ^a^	4.07 ± 0.01 ^de^	28.94 ± 1.28 ^ab^	30.18 ± 1.94 ^b^	25.2 ± 1.8 ^ab^
**C1237**	94.50 ± 0.74 ^a^	23.62 ± 0.60 ^abc^	4.11 ± 0.02 ^de^	23.70 ± 1.20 ^cd^	34.35 ± 1.37 ^a^	16.7 ± 1 ^d^
**C1238**	95.21 ± 0.95 ^a^	24.49 ± 1.31 a	4.27 ± 0.02 ^bc^	26.35 ± 1.47 ^bc^	29.99 ± 1.00 ^b^	17.1 ± 0.1 ^cd^
**C1239**	95.56 ± 0.22 ^a^	20.01 ± 0.52 d	4.37 ± 0.02 ^b^	26.54 ± 1.06 ^bc^	30.61 ± 0.58 ^bc^	19.3 ± 0.1 ^c^
**C1240**	95.61 ± 0.31 ^a^	20.22 ± 0.77 cd	4.55 ± 0.04 ^a^	26.12 ± 1.54 ^bc^	31.43 ± 0.78 ^ab^	23.2 ± 0.3 ^b^
**C1241**	95.31 ± 0.30 ^a^	21.05 ± 1.33 ^bd^	4.25 ± 0.05 ^c^	29.37 ± 0.26 ^ab^	30.54 ± 1.37 ^bc^	25.2 ± 1.7 ^ab^
**C1243**	95.48 ± 0.20 ^a^	24.04 ± 1.70 ^ab^	4.28 ± 0.06 ^bc^	30.75 ± 1.26 ^a^	26.39 ± 0.78 ^d^	26.5 ± 0.5 ^a^
**C1244**	95.61 ± 0.09 ^a^	24.57 ± 0.94 ^a^	4.14 ± 0.06 ^cd^	26.64 ± 1.70 ^bc^	33.62 ± 0.20 ^ac^	23.7 ± 0.3 ^b^

Parameters include dry matter (%DM), crude fiber (%CF), ash (%A), crude protein (%CP), oil (%O) and glucosinolates (GLS). Statistically significant distinctions for each measured parameter are denoted by varying letters, as determined by Tukey’s test (p < 0.05).

## 4 Discussion

Rotation and intercropping are both agricultural practices aimed at maximizing yield and sustainability while minimizing the negative impacts on the environment offering several benefits: maintain soil fertility and efficient use of resources, pest and disease management and weeds control contributing to Climate‐smart agricultural (CSA) (Tabe-Ojong et al., 2024). However, despite the undeniable usefulness of intercropping, this practice is currently underutilized in developed countries, while in developing countries, it remains fundamental for the food security in smallholders (Santalucia and Sibhatu, 2024). There are several reasons why intercropping is less commonly used in developed countries: specialization and monoculture, market demands, land ownership and policies, and a lack of specific breeding programs, particularly regarding cover crops (Rubiales et al., 2023). In recent years, camelina has been gaining considerable interest from the scientific community due to its ability to thrive in conditions with limited water resources and poor-quality soils, making it a sustainable alternative to traditional oilseed crops, due also to the short life cycle (85–100 days for spring varieties, 190–210 for winter varieties) (Ahmad et al., 2022; Pascual et al., 2024). Researchers are currently exploring camelina as a biofuel crop and a new source of protein and oil due to its high seed oil content of about 40%, rich in polyunsaturated fatty acids (including 30–40% α-linolenic acid, 15–25% linoleic acid, 15% oleic acid, and approximately 15% eicosenoic acid) (Rodríguez-Rodríguez et al., 2021; Arshad et al., 2022). Moreover, the residue from seed pressing, known as cake, can be utilized in animal diets as a source of proteins and oils (Hajiazizi et al., 2024). Despite its potential, the presence of glucosinolates (GLS), sulfur molecules involved in plant defense, limits camelina’s use in feed and food applications (Russo and Reggiani, 2012; Russo et al., 2014; Murphy, 2016). Nonetheless, camelina exhibits disease-suppressing properties, thanks to glucosinolates that exhibit fungicidal, nematocidal and bactericidal activities (Arora and Kaushik, 2003). In fact, several studies have also indicated that camelina as a cover crop mitigates root diseases and improves the growth and yields of various crops, highlighting its potential as a sustainable and integrated pest management strategy in agriculture, making it emerge as a promising cover crop with weed control potential (Acharya et al., 2020; Ghidoli et al., 2023b).

For example, in a study conducted in the USA, sowing winter camelina into standing maize and soybean crops prior to their harvest enhanced soil properties and increased overall system productivity (Berti et al., 2017). In another study conducted in semiarid Mediterranean areas, the potential of camelina to reduce the presence of corn poppy and its seed production was demonstrated as a valuable tool for Integrated Weed Management (IWM), using barley as the main crop (Codina-Pascual et al., 2022). Additionally, intercropping spring-sown field peas with camelina significantly suppressed weed coverage compared to monocropped pea plots (Saucke and Ackermann, 2006).

Despite the great potential of this crop, there have been few breeding efforts aimed at studying and improving some characteristics of this plant to enable its widespread use in rotation or intercropping with other crops (Ghidoli et al., 2023a). In particular, the genetic improvement of this crop, aimed also at intercropping purposes, should shorten the vegetative cycle, increase seed size (to facilitate mechanized sowing and harvesting), thus making spring utilization easier as well.

With the aim to develop a new camelina variety, we started a breeding program based on the study of a survey of two main cluster groups (spring and winter varieties) by using genetic tools. The first molecular analyses were conducted by using 16 SSRs reported from the work of Manca et al. (2014) on six winter varieties and five spring varieties (Manca et al., 2014). The results obtained allow us to discriminate the two different habits as reported in **Figure 1**. This result was confirmed by using the genotyping by sequencing technique on two spring varieties (C1238 and C1243) and two winter varieties (C1237 and C1233) (**Figure 2**) exploring the variability at a definitely higher genome-wide resolution. Moreover, the data collected through the GBS analysis might be further analyzed to identify variants affecting genes possibly correlated with seasonal differences. Our results confirmed the study conducted on various members of the camelina genus by Chaudhary et al. (2020), in which GBS was employed to analyze 193 camelina accessions belonging to different species. The results showed that camelina species were represented by two clusters, the spring-type varieties and the winter-type varieties, in particular, the winter-type camelina lines may probably, from a phylogenetic point of view, be derived from a hybridization between *C. sativa* and *C. microcarpa* (Chaudhary et al., 2020). This last hypothesis could explain the strong similarity of the F1 hybrid (**Figures 3B, C**) generated by our breeding program (started by crossing spring x winter varieties) and the results obtained by Tepfer et al. (2020) by crossing a *C. sativa* spring variety and *C. microcarpa* (Tepfer et al., 2020). We decided to cross a spring (C1243) with a winter (C1233) variety to maximize the genetic diversity selectable in our breeding program, based on bulk method selection for yield, earliness and weight of 1000 seeds. As reported before, the F1 hybrid showed in the first stage of development an intermediate characteristic of the two habits of varieties (**Figure 3B**) and a high hybrid vigor (shrubby habit, taller and more branched plants and woody stems with larger diameters) was shown on reaching maturity (**Figure 3C**). The results obtained suggest that a utilization of F1 seeds could be possible, in the near future, by using a male sterile mutation that so far, to our knowledge, has not yet been found. The new variety obtained (C1244) was cultivated in winter for two consecutive years with all the varieties under study reported in **Table 1**.

The comparison of all the varieties allowed us to collect the data regarding the yield (kg/ha), the number of plants per m^2^ and the weight (g) of 1000 seeds reported in **Table 3**. In our work, we measured yields ranging from 3393 kg/ha to 1670 kg/ha (**Table 3**), consistent with previously reported data for the Northern Italy region. In fact, Masella et al. (2014) reported a yield ranging from 1200 to 3300 kg/ha studying different varieties. Camelina, thrives in various soil types and grows particularly well in cool semi-arid climates. While it can endure drought, it may hinder delicate growth stages like flowering (Berti et al., 2016). However, the most abundant seed yields have been observed in Mediterranean climates where camelina cultivation is gaining more and more interest especially in Italy (Berti et al., 2016; Estakhr and Ranjbar, 2021).

Evaluating W1000, we found that on average the seed-size of spring varieties is higher compared to that of winter varieties (W1000 means respectively 1.31 and 1.15g) as shown in **Table 3**. This result is in agreement with a previous work, where the winter and spring genotypes were differentiated by most seed shape descriptors and in particular by seed weight (Wiwart et al., 2019).

The new variety C1244 recorded a weight of 1000 seeds statistically higher than the two parents (**Figure 5C**), as well as the highest recorded in both agronomic years (**Table 3**). The increase in seed weight obtained in the selection process is a positive parameter as for crops with small seeds this can lead to facilitating both sowing and harvesting operations.

A further trait of interest obtained from the output of our breeding program was the decreasing in the crop cycle (**Figure 5D**). C1244, thanks to the selection, flowers earlier than all the spring varieties under study. This trait holds significant importance in intercropping and rotation systems, especially in dry regions (Zubr, 1997; Berti et al., 2016; Neupane et al., 2022). Additionally, apart from its role in crop rotations, C. sativa can offer various ecosystem services, such as preventing soil erosion and nutrient runoff during early spring (Gesch, 2013) and providing habitat for pollinators (Eberle et. al., 2015). Although C. sativa yields relatively modest amounts of biomass compared to larger crops, its crop residues can enhance soil water absorption capacity, which is particularly beneficial in dry soil regions (Lohaus et al., 2020).

Taking together all the agronomic traits present in **Table 3** and flowering data (data not shown) for all the varieties, PCA multivariate analysis clustered the two main camelina growing season habits, confirming again the genetic data (**Figure 6**).

Bromatological analyses, concerning dry matter, crude fiber, ash, crude protein, and oil, and glucosinolate quantification were carried out on the collected seeds (**Table 4**). The bromatological and glucosinolate analyses were conducted with the aim of assessing camelina’s suitability for use in both feed and food applications. A primary concern lies in the presence of glucosinolates (GLS), which represents a significant limitation for these applications. However, these molecules play a role in plant defense mechanisms, and, as noted by Ghidoli et al. (2023b), their occurrence can be attributed to the allelopathic properties inherent in camelina (Quéro et al., 2016; Hofmann et al., 2023). A positive correlation (r = 0.69; p = 0.013) was observed between protein and glucosinolate content (**Figure 8**), and the same result was obtained in the previous study (Ghidoli et al., 2023c), in which two hypotheses were proposed to explain the correlation. The first hypothesis suggests that the presence of sulfur-containing amino acids, from which glucosinolates are synthesized, could account for this correlation, given the sulfuric nature of glucosinolates (Ghidoli et al., 2023b). The second hypothesis takes into consideration the 2008 study by Williams and colleagues, highlighting epithiospecifier proteins (ESPs) as non-catalytic cofactors of myrosinase—the enzyme responsible for catalyzing the hydrolysis of glucosinolates (Williams et al., 2008). According to this hypothesis, the correlation between protein and glucosinolate content may be attributed to the influence of ESPs on the glucosinolate levels (Lambrix et al., 2001; Wittstock and Burow, 2007; Williams et al., 2008; Kuchernig et al., 2012). Finally, also in this case, as shown in **Figure 9**, PCA obtained using bromatological traits and glucosinolates content confirmed the presence of two distinct camelina agronomic behaviors.

Recognizing the significance of genetic diversity is crucial for formulating a resilient breeding strategy that involves identifying and integrating essential variations to enhance future crop improvement efforts. In this study, seed size emerges as a crucial factor influencing both a high oil yield and the improvement of all mechanized agricultural operations. The newly developed variety, C1244, demonstrated the most favorable combination of agronomic and chemical parameters. This suggests that classical breeding programs can prove beneficial in enhancing this species, even in the face of its limited genetic variability. Finally, the new variety, which is characterized by high yield, large seeds, and a short vegetative cycle, could be used both as a winter cover crop and in spring intercropping, particularly with legumes and cereals, to enhance the sustainability of these agricultural crops and improve European farming systems.

## Data availability statement

The raw data supporting the conclusions of this article will be made available by the authors, without undue reservation.

## Author contributions

MG: Visualization, Writing – original draft, Software, Methodology, Investigation, Formal analysis, Data curation, Conceptualization. FG: Supervision, Writing – review & editing, Software, Methodology, Formal analysis, Data curation. SD: Writing – review & editing, Software, Methodology, Formal analysis, Data curation. SF: Writing – review & editing, Software, Methodology, Formal analysis, Data curation. ML: Investigation, Writing – review & editing, Supervision, Methodology. EC: Writing – review & editing, Supervision, Methodology, Formal analysis, Data curation. AS: Writing – review & editing, Supervision, Methodology. LR: Writing – review & editing, Supervision, Methodology. SP: Writing – review & editing, Visualization, Validation, Supervision, Resources, Project administration, Investigation, Funding acquisition, Conceptualization.
