# Supplementary material for: Genetic study of Camelina sativa oilseed crop and selection of a new variety by the bulk method
Source: Front Plant Sci. 2024 May 28;15:1385332. doi: 10.3389/fpls.2024.1385332 (PMC11165348; doi:10.3389/fpls.2024.1385332)

Supplementary Material

Genetic study of *Camelina sativa* oilseed crop species and selection of a new variety by bulk method

Martina Ghidoli^1^, Filippo Geuna^1^, Stefano De Benedetti^2^, Sara Frazzini^3^, Michela Landoni^4^, Elena Cassani^1^, Alessio Scarafoni^2^, Luciana Rossi^3^ and Salvatore Roberto Pilu^1*^

*** Correspondence:**Salvatore Roberto Pilu
[salvatore.pilu@unimi.it](mailto:salvatore.pilu@unimi.it)

| **Table S1**. Camelina sativa microsatellite loci identified in Calena variety. (Modified from Manca et al., 2012) | | | |
| --- | --- | --- | --- |
| **Name** | **Primer sequences** | **Range size (bp)** | **Ta (°C)** |
| P6E4 | F: CCGTGGAGGAAGATGATTGAGA  R: TGCCCCAAATCCAAGTTCG | 303-327 | 54 |
| LIB19 | F: AACCGTGAACCTACTACAGAGAAAGA  R: TCTGGGATTTACCGAACCAA | 115-151 | 54 |
| P4B3 | F: ATGGACGACGAGAGGTGTGA  R: CGATCTGAGCAGGCTCCATT | 210 | 55 |
| P3C3 | F: TTGGCCATTGCTTGGGTGTA  R: ACTGGATTCCGTGCCTTGGT | 178-222 | 55 |
| P3H4 | F: CCAGACAAACACACGAAGCCGAAT  R: CTGGGCGTAGGGTTTTAGTCCATT | 200-204 | 58 |
| P4C2 | F: CGTCTCCCGTTGTTCCAAGT  R: TCACAAATCACCAATTCCCAAG | 174-186 | 58 |
| P4C7 | F: GCCGTCTCCAAAGGCAGAAG  R: GCAGCTCTCATCCTTGGTTTTG | 257-276 | 58 |
| P4E6 | F: CACATCCAAAAGCTCTCTTTCTCTT  R: GACAGCGATGGTTTTAAGAAAGTTGA | 189-226 | 58 |
| P4C11 | F: TGAGCCTTTATAGAAGTTTCGGAACAA  R: TGCTGATGTCACACGGAGGA | 181-229 | 58 |
| P4H3 | F: TCTGTGTCCGCATGGGAAGT  R: CACGTAAGCAATCAAATCCCTCTC | 219-225 | TD1 |
| P6C2 | F: CATGCTCTACTACCAAACCCAACA  R: TTGATCCTCTCTCAAGGTATCTCTTTC | 134-170 | TD1 |
| P7A4 | F: CCCAGGAAGCCACAGACA  R: GCATAAATCGCAAATGAAAGAGTAG | 146-179 | TD2 |
| P7D9 | F: CGGATAGATGACCCAGATCCA  R: GTGGAGCATTCCATTCAGTCAG | 170-182 | TD2 |
| P7D4 | F: TGGTCCCCTGACGAAAGAA  R: CCTCTGCCACAACCTTCACA | 129-173 | TD2 |
| P7C2 | F: ATCCCAACATGCTCACAAGG  R: GGACATCAGACAACACACGAGA | 174-210 | TD2 |
| P7A8 | F: GCGGTGGAGGAAAGGTTTTA  R: CCGGCCCAATATCTCTGATG | 176-206 | TD2 |

| Table S2. Accumulated precipitation and mean monthly temperatures relating to the two years of winter cultivation. | | | |
| --- | --- | --- | --- |
| Year | **Month** | **Mean Monthly Temperature (°C)** | **Monthly Rainfall (mm)** |
| 2021-2022 | October | 14.1 | 52 |
|  | November | 9.5 | 165.6 |
|  | December | 3.1 | 46 |
|  | January | 3.2 | 28 |
|  | February | 7.6 | 16 |
|  | March | 9.1 | 9.8 |
|  | April | 14.4 | 25 |
|  | May | 22.5 | 62 |
|  |  |  |  |
|  | Total |  | 404.4 |
|  | | | |
| 2022-2023 | October | 18.4 | 9.4 |
|  | November | 9.9 | 101.6 |
|  | December | 6.0 | 97.6 |
|  | January | 5.9 | 77 |
|  | February | 7.3 | 3.6 |
|  | March | 11.8 | 21 |
|  | April | 14.7 | 42.6 |
|  | May | 20.2 | 69.6 |
|  |  |  |  |
|  | Total |  | 422.4 |

**Table S3.** Tukey’s test post-hoc analysis regarding Two-way ANOVA (**Table 2**). Factor takes into consideration yield (Y). Pink boxes highlight p < 0.05.


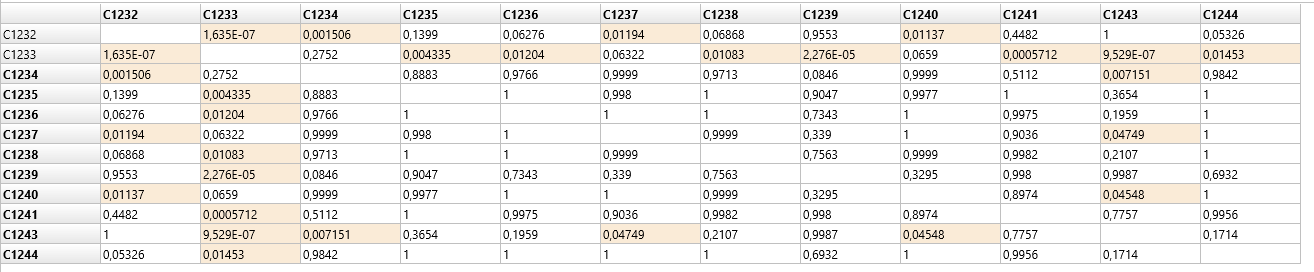


**Table S4.** Tukey’s test post-hoc analysis regarding Two-way ANOVA (**Table 2**). Factor takes into consideration number of plants per m^2^ (NP). Pink boxes highlight p < 0.05.


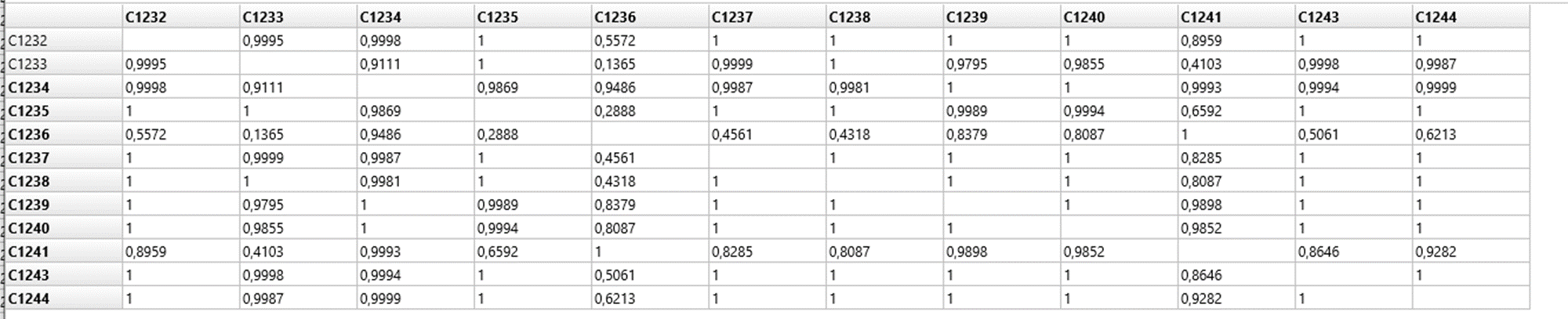


**Table S5.** Tukey’s test post-hoc analysis regarding Two-way ANOVA (**Table 2**). Factor takes into consideration weight of 1000 seeds (W1000). Pink boxes highlight p < 0.05.


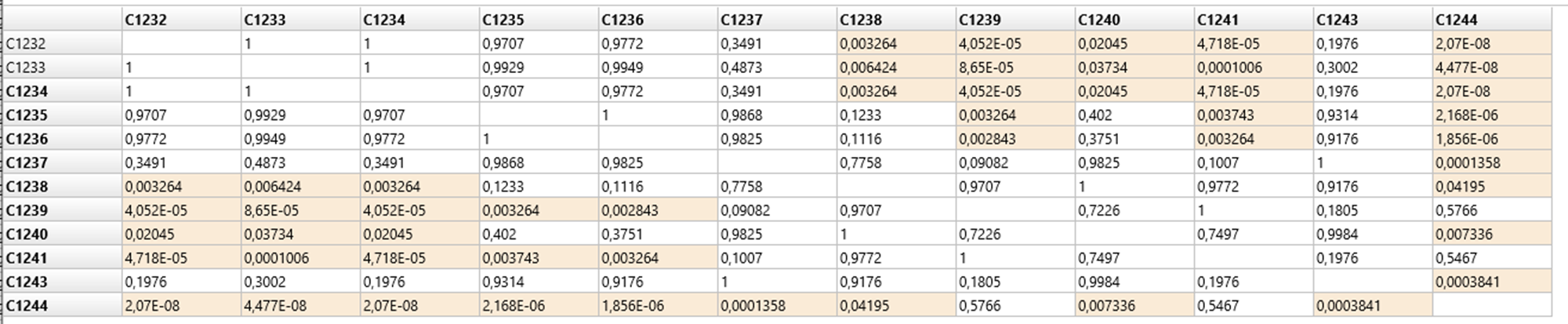

Supplement: Supplementary file 1 [file Table_1.docx]
